# Theoretical Prediction of Disrupted Min Oscillation in Flattened *Escherichia coli*

**DOI:** 10.1371/journal.pone.0139813

**Published:** 2015-10-12

**Authors:** Jeff B. Schulte, Rene W. Zeto, David Roundy

**Affiliations:** Dept. of Physics, Oregon State University, Corvallis, Oregon, United States of America; University of California San Diego, UNITED STATES

## Abstract

The dynamics of the Min-protein system help *Escherichia coli* regulate the process of cell division by identifying the center of the cell. While this system exhibits robust bipolar oscillations in wild-type cell shapes, recent experiments have shown that when the cells are mechanically deformed into wide, flattened out, irregular shapes, the spatial regularity of these oscillations breaks down. We employ widely used stochastic and deterministic models of the Min system to simulate cells with flattened shapes. The deterministic model predicts strong bipolar oscillations, in contradiction with the experimentally observed behavior, while the stochastic model, which is based on the same reaction-diffusion equations, predicts more spatially irregular oscillations. We further report simulations of flattened but more symmetric shapes, which suggest that the flattening and lateral expansion may contribute as much to the irregular oscillation behavior as the asymmetry of the cell shapes.

## Introduction

It is vital that during the process of bacterial cell division a cell avoid minicelling, or splitting into daughter cells with lopsided volumes. Instrumental to this process in *Escherichia coli* is a long FtsZ polymer chain that develops near the cell wall in the center region of the cell, helping dictate the plane of division [1, 2]. Previous experimental studies have shown that the MinC protein, known to inhibit the FtsZ polymer [3], exhibits regular pole to pole oscillatory behavior between both ends of the wild-type pill-shaped cell. It thus has a higher time averaged concentration in the cell poles than in the center region, which aides in preventing the FtsZ from developing in the wrong region. The MinC is recruited to these poles by MinD, which itself interacts with another protein, MinE, in a system which exhibits pole-to-pole oscillatory behavior [4–9].

Previous experimental studies have shown that the MinD protein system is capable of exhibiting oscillations in round shapes [10, 11] as well as in connected three pronged tube shapes [12], in which the oscillations seem to seek out the extreme poles in the cell. Männik *et al*. have recently shown that there are limitations to this robust capability to oscillate [9, 13, 14]. They have experimentally forced *E. coli* cells into microfabricated channels of thickness less than that of the natural diameter of the cells, in which they area able to grow and divide. Upon entering the channels, the cells undergo a mechanical deformation in which they both widen and lengthen within the plane of the channel. This deformation results in very wide (they can reach widths of over 5*μ*m) flattened cells that when viewed from the top down have irregular and asymmetric shapes. While these cells are still able to divide into surprisingly equal volumes, the MinD oscillations in these cells are spatially irregular. Seen with fluorescent microscopy, the MinD maximize in multiple locations within the cell, in a seemingly random sequence. These experiments allow for an opportunity to test models of the MinD system against more extreme cases than have been seen thus far. An additional interesting question is whether the irregularity observed by Männik *et al*. indicates that the MinD system can become chaotic (as in e.g. Ref. [15]), or if this irregularity is the result of a stochastic but nonchaotic process.

A number of models of the MinD protein system have been developed that accurately describe its basic oscillatory nature. Early models involved free proteins that affect each others’ rates of diffusion and membrane attachment, but do not combine into compound states [16]. Kruse extended this model by incorporating recruitment and clustering of MinD [17]. In 2003 Huang improved upon Kruse’s approach with a simple and very successful simulation model that incorporates MinD-MinE combination, ATPase hydrolysis, and MinD membrane attachment that exhibits accurate MinD oscillations in cylindrical cells [7]. In this model cytoplasmic MinD is recruited to the membrane by MinD that is already clustered there (following observed non-linear attachment of MinD on the cell membrane [18, 19]), and is stationary once attached.

Several models [20, 21] modify Huang’s original model to more accurately reflect experimental findings, which were published after his 2003 paper [22, 23]. These models introduce diffusion along the membrane that is two to three orders of magnitude slower than that within the cytoplasm, and increase the rate of cytoplasmic diffusion by a factor of about five, reflecting an experimental measurement of these parameters [22]. They also increase or assume instantaneous the nucleotide exchange rate [20]. In addition, they revise the process of MinD recruitment to the membrane, removing recruitment of MinD to the membrane by MinD-MinE complexes, behavior that has not been observed experimentally. The model of Bonny *et al*. further more allows MinE to remain independently attached to the membrane after disassociating with MinD [20].

There are only on the order of a thousand MinD proteins in a given cell, which is fewer than the number of grid points used in our simulations, suggesting that the deterministic continuum description–which requires fractional numbers of proteins at each grid point—may break down, and that stochastic behavior may play a significant role. To treat this, there have been a number of applications of the Huang 2003 model that stochastically simulate the same reaction-diffusion equations [8, 11]. These studies largely confirm the results of Huang’s deterministic model when applied to the wild-type, pill-shaped phenotype. However, the stochastic models are slightly more successful in predicting experimentally observed oscillations in round cell phenotypes [11, 24], and they enable prediction of fluctuations in the predicted behavior [25], including the observation of stochastic switching in short cells instead of a transition to static behavior as would be predicted from a deterministic model [26]. Both deterministic and stochastic models are widely used throughout systems biology [27–31], and have unique advantages and limitations. The deterministic approach has an advantage in providing a simple prediction of average behavior, while the stochastic approach enables prediction of fluctuations from that mean, and reduces sensitivity to initial conditions.

In this paper we use Huang’s original 2003 model [7], which has been widely used [8, 11, 24, 32] in both deterministic and stochastic variants, to study flattened cells of 0.4*μ*m thickness that are similar to those observed by Männik *et al*.[9]. This allows us to test the performance of this important model in more extreme situations than has been done previously, furthering our understanding of how and to what extent its predictions fail.

## Materials and Methods

### Model and Cell Shapes

**Fig 1:**
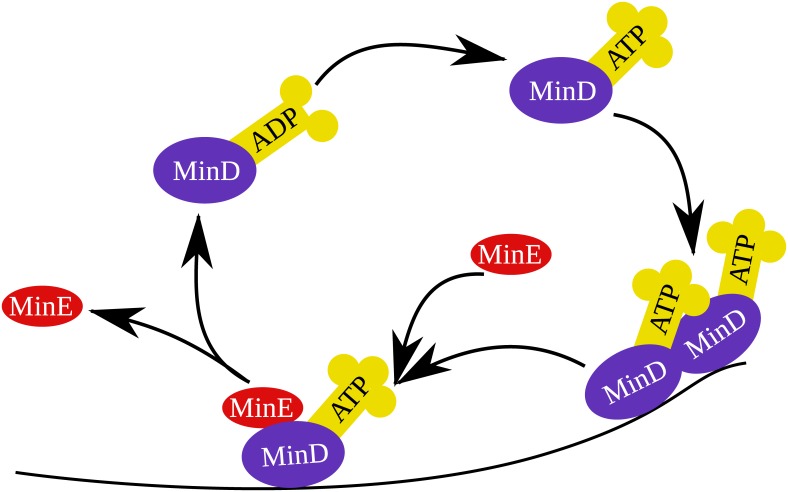
Reactions included in the Min system model of Huang *et al*., which is used in this work [7].

We implement the reaction-diffusion model of Huang *et al*.[7]. Fig 1 shows the reaction process. The cytoplasmic MinD:ADP complex undergoes nucleotide exchange and is changed into the MinD:ATP complex. This will naturally diffuse and attach to the cell membrane. A cytoplasmic MinE will attach to the wall bound MinD:ATP complex and after a time will activate ATP hydrolysis. This breaks up the complex, releasing MinE, phosphate, and MinD:ADP back into the cytoplasm. The MinD:ADP will undergo nucleotide exchange and begin again the cyclic process. The model is defined by a set of five reaction-diffusion equations:

$$\frac{\partial \rho_{D:ADP}}{\partial t}=D_{D}\nabla^{2}\rho_{D:ADP}-k_{D}^{ADP\to ATP}\rho_{D:ADP}+\delta \left(d_{w}\right)k_{de}\sigma_{DE},\;$$ (1)

$$\frac{\partial \rho_{D:ATP}}{\partial t}=D_{D}\nabla^{2}\rho_{D:ATP}+k_{D}^{ADP\to ATP}\rho_{D:ADP}-\delta \left(d_{w}\right)\left[k_{D}+k_{dD}\left(\sigma_{D}+\sigma_{DE}\right)\right]\rho_{D:ATP}$$ (2)

$$\frac{\partial \rho_{E}}{\partial t}=D_{E}\nabla^{2}\rho_{E}+\delta \left(d_{w}\right)k_{de}\sigma_{DE}-\delta \left(d_{w}\right)k_{E}\sigma_{D}\rho_{E}$$ (3)

$$\frac{\partial \sigma_{D}}{\partial t}=-k_{E}\sigma_{D}\rho_{E}+\left[k_{D}+k_{dD}\left(\sigma_{D}+\sigma_{DE}\right)\right]\rho_{D:ATP}$$ (4)

$$\frac{\partial \sigma_{DE}}{\partial t}=-k_{de}\sigma_{DE}+k_{E}\sigma_{D}\rho_{E}\;$$ (5)

where *ρ* is cytoplasmic protein density (proteins/*μ*m^3^), *σ* is membrane bound density (proteins/*μ*m^2^), $D_{D}$ and $D_{E}$ are the diffusion constants for MinD and MinE, respectively, $k_{D}^{\text{ADP}\to \text{ATP}}$ is the rate of conversion from MinD:ADP to the MinD:ATP complex, *k*_*D*_ is the rate of MinD:ATP attachment to the membrane when no protein is already attached there, *k*_*dD*_ is the increase of this rate when MinD:ATP is present on the membrane, *k*_*de*_ is the rate of hydrolysis of the MinD:MinE:ATP complex, *k*_*E*_ is the rate of cytoplasmic MinE binding to membrane bound MinD:ATP complex, and *d*_*w*_ is the distance from the point in space to the closest wall. The Dirac delta function *δ*(*d*_*w*_), which we need to describe the location of the membrane, has units of *μ*m^−1^ and is zero everywhere except at the wall. Eqs 4 and 5 are only relevant at the membrane because the membrane-bound density values have no meaning in the cytoplasm.

Our diffusion and reaction rates are shown below. We are interested primarily in the effect of cellular size and shape on the protein oscillations, so we use Huang’s parameter values [7].

$$\begin{array}{c} D_{D}=D_{E}=2.5\mu {\text{m}}^{2}/\text{sec}\\ k_{D}^{\text{ADP}\to \text{ATP}}=1/\text{sec},k_{D}=0.025\mu \text{m}/\text{sec}\\ k_{dD}=0.0015\mu {\text{m}}^{3}/\text{sec},k_{de}=0.7/\text{sec}\\ k_{E}=0.093\mu {\text{m}}^{3}/\text{sec}. \end{array}$$

Huang’s simulations use total MinD and MinE concentrations of 1,000/*μ*m and 350/*μ*m, respectively, in a cylindrical cell of radius 0.5*μ*m, and in our (non-cylindrical) cells we use the same number of proteins per unit volume. These concentration values are 1273*μ*m^−3^ and 446*μ*m^−3^, respectively. We have written our own simulation platform and membrane creation tools from scratch, instead of using existing available software used in previous studies [8, 11, 20, 21]. Our simulations take place within a three-dimensional Cartesian grid that has a grid spacing of .05*μ*m, and our cell shapes are specified as the zero of an analytic function which can generate the geometry presented in this paper. This code is publicly available on github [33], including scripts that generate the simulation data used in this paper.

We have performed both a numerical, deterministic model simulation that is spatially and temporally discrete, and a stochastic simulation that is spatially discrete but continuous in time. Our stochastic model follows the work of Kraus [34] which in turn follows a method introduced by Gillespie [35].

We mean to investigate the geometric limits of the Min system oscillations as observed by Männik *et al*.[13], so we have modeled the Min system in several cell shapes and sizes. Here we present a selection of these, beginning with naturally occurring pill-shaped cells, followed by a number of flattened out shapes which reflect the experiments of Männik *et al*., in which bacteria are confined within thin slits. These slits are fabricated with a width of .25*μ*m, but they are coated with a PDMS lining that the cells are able to deform, raising uncertainty in the actual cell thickness. Tests with pure silicon, non-deformable slits show that the smallest thickness for which cells are able to penetrate is .4*μ*m [9, 13]. We therefore assume that the PDMS slits have been deformed to this thickness and simulate flattened cells with a thickness of .4*μ*m. Viewed from the top down the cells have the shapes described below and viewed from the side they have at their edges a semicircular cross-section (one may imagine the shape of a pancake). In this paper we focus on four specific flattened cell shapes. Two of these shapes replicate those published by Männik *et al*., and the other two are ‘stadium’ shapes that respectively have the same aspect ratio, thickness, and volume as the two cell shapes experimentally observed by Männik *et al*.[13]. Viewed from the top down, these stadium shapes appear as rectangles with semi-circular end caps on the long axis ends.

Huang *et al*.[7] have performed a linear stability analysis on a cylindrical model which shows an upper limit on a steady state solution of a 2*μ*m half wavelength. Cells with dimensions longer than this spontaneously develop spatial oscillations, while cells that are shorter relax into a homogeneous steady state. We have performed a similar linear stability analysis on an infinite slab with a thickness equal to that of our flattened cells (0.40*μ*m). We first solve the model’s five differential equations for the steady state solution, under the constraint that the total number MinD and MinE molecules matches our simulation. We write down as a matrix the linear response of the time derivatives of the density to small, spatially harmonic density perturbations around the steady-state density. The system is stable at this wavelength provided all eigenvalues of the matrix are negative, indicating that all perturbations with this wavelength will decay. We find the stability limit by searching for the largest wavelength at which the system is stable. Through this method we arrive at an upper half wavelength stability limit of 1.56*μ*m for our 0.40*μ*m flattened cells. As expected, when decreasing the lengths and widths of our simulated flattened cells so that the longest distance across the cell is less than this length, the cells stop exhibiting any oscillatory behavior. The deterministic model relaxes into a motionless state and the stochastic model exhibits random fluctuations without spatial oscillations.

## Results and Discussion

### Naturally Occurring Pill Shaped Cells

We begin with the naturally occurring pill cell shape. We piece this shape together as a cylinder with hemispherical end caps. This shape follows the early simulations of Huang *et al*. but differs in that we have added the end caps for a more natural shape, expecting similar results.

**Fig 2:**
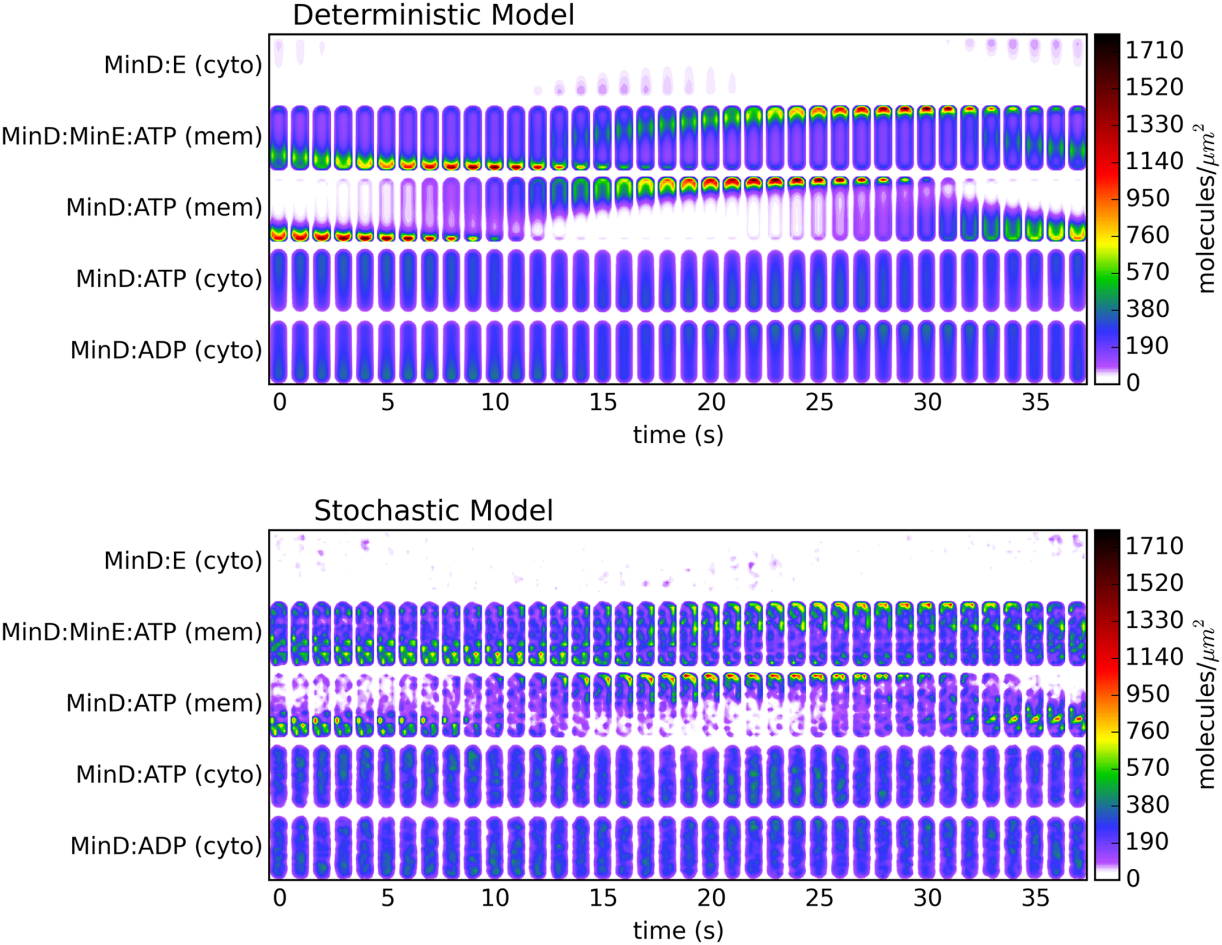
Images of the concentration of each protein species in a natural pill-shaped bacterium at one-second time intervals. The upper plots shows results from the deterministic model and the lower shows results from the stochastic model. The order of frames is such that individual MinD proteins begin at the bottom of the plot (in the MinD:ATP state in the cytoplasm), and progress upward until they reach the MinE:MinD:ATP membrane-bound complex. At that point, they will spontaneously dissociate into cytoplasmic MinE (the top row) and the starting state of cytoplasmic MinD:ADP.

Fig 2 shows a series of color plots of the density of proteins at each stage of the reaction cycle. Deterministic simulation data is shown above and stochastic simulation data is shown below. The cells shown are 4*μ*m in length, measured from end to end. We have ‘smeared out’ the stochastic concentrations in a manner meant to reproduce the images shown by diffraction limited fluorescence microscopy. We do so using the two dimensional Gaussian approximation developed by Zhang *et al*.[36]. In this approximation we use a numerical aperture value of 1.3, which is the same as used by Männik experimentally, and a wavelength of 650*nm*. Each frame is 2.5 seconds ahead of the last, and each image shows the concentration of a given state of protein (of the five described in the reaction model) summed over the coordinate normal to the page.

Fig 2 begins about 300 seconds into the simulation and shows one period of oscillation. At *t* = 0 there is a high concentration of MinD:ATP that has accumulated on the membrane at the bottom of the cell. An important aspect of Huang’s model is that the MinD is attracted to and sticks to the membrane nonlinearly: as it accumulates there it begins to ‘recruit’ other MinD that is diffusing in the cytoplasm nearby, causing peaks in concentration to build up on the walls. Meanwhile, the MinE creeps downward as it reacts with the membrane-bound MinD, forming the MinD:MinE:ATP complex, then breaks it apart and diffuses downward a bit more before it again reacts with membrane-bound MinD. This process can be seen in the form of the well-known “MinE rings” (actually, MinE bound to MinD on the membrane). These rings are visible in the deterministic model plots as a green band on the walls from 0 seconds to 4 seconds (and later from 15 to 23 seconds). The appearance of these rings in the deterministic model alongside their less obvious appearance in the stochastic model highlights an advantage of the deterministic model: the idealization of deterministic data allows one to see patterns in the averaged behavior that might otherwise be missed. In the stochastic model the MinE still exhibits higher concentrations in the same regions throughout the process, but what would ideally be a “ring” pattern is instead an asymmetric collection of maxima, which would become a ring after phase-locked averaging.

During the formation of these rings, cytoplasmic MinE is diffusing in the upper portion of the cell and will naturally progress downward, where there is membrane-bound MinD to react with, leading to a depletion of MinE in the upper portion of the cell. As the MinE ring converges upon the lower end of the cell, MinD that has been released is able to diffuse upward, past the ring, while still in its MinD:ADP state and unable to bind to the membrane. After it undergoes nucleotide exchange, resulting in MinD:ATP, it is ready to accumulate on the walls in at the top of the cell, where the MinE has been depleted. This can be seen in seconds 10 through 20 in both models, followed by the subsequent MinE ring formation and movement upward (beginning the same process in the opposite direction) that can be seen in seconds 15 through 23.

Both Fig 2 and its associated movies (see supplementary material), show that the stochastic model exhibits a “starry night” effect characterized by spatially fixed points of protein density build up, as recruitment leads to clusters of MinD forming on the wall and then subsequently dissipating. This result, which is not observed in experiments, stems from the omission of diffusion along the membrane from Huang’s model, which would allow these recruitment clusters to spread out. Experiments have confirmed that diffusion along the membrane does occur, albeit with a rate two orders of magnitude lower than that in the cytoplasm [22]. The ‘stars’ in the effect typically last for around 10 seconds. The membrane-bound proteins will diffuse by a distance of around one micron during this time, which is enough to spread out across the polar section of the one micron diameter wild-type pill cells. As we see below, the flattened, irregular cells are large enough that this one micron blurring might not completely eliminate this ‘starry night’ effect.

**Fig 3:**
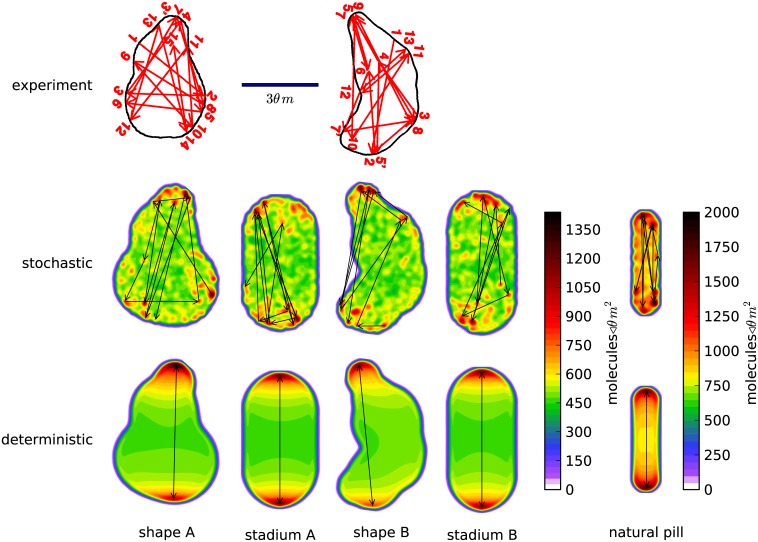
Temporal correlation function of the total MinD found in two opposite polar regions of the pill-shaped cell, shown against the correlation time. Data for both the the deterministic and stochastic models are shown. The stochastic model shows an oscillation period of 39.5 seconds and coherence time of 307 seconds. The correlation functions are scaled to have the same initial value.

We also analyze the temporal periodicity of the system, which we quantify with the temporal correlation function of the total MinD found in two opposite polar regions. This correlation function, which is displayed in Fig 3, is given by

$$C\left(\tau \right)\propto \int \left(N_{top}\left(t\right)-{\bar{N}}_{top}\right)\left(N_{bottom}\left(t+\tau \right)-{\bar{N}}_{bottom}\right)dt$$ (6)

where *N*_*top*_(*t*) and *N*_*bottom*_(*t*) are the total MinD proteins in the top and bottom thirds of a cell. These plots help us in studying the periods and the regularity and stability of oscillations. These curves are well fit with a simple decoherence model, given by the equation

$$C\left(\tau \right)=-\cos(\frac{2\pi \tau }{T})e^{-\frac{\tau }{\tau_{c}}}$$ (7)

where *T* is the period and *τ*_*c*_ is the coherence time. In the case of the stochastic model the pill exhibits coherence times of about 8 periods, indicating that the system exhibits periodic oscillation, with some stochastic irregularity in the period. In contrast, in the deterministic model the pill-shaped cell exhibits complete coherence, indicating that its behavior is perfectly periodic.

### Comparison of Experimental and Stadium Shapes

**Fig 4:**
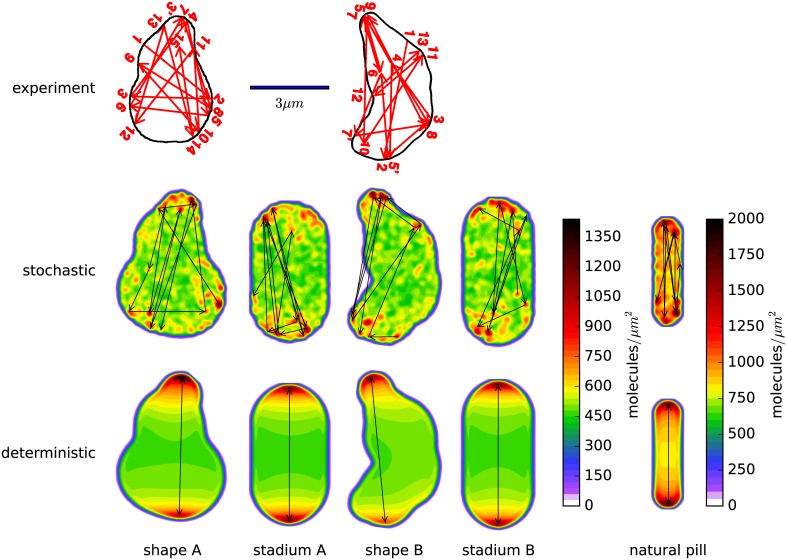
Arrows depicting successive maxima in space and time overlayed on a color plot of the total MinD density averaged over the same time period. The simulation time covered for the flattened cells is 350 seconds, which is the same period of time depicted in the experimental data plots of Männik *et al*.[13]. For the wild-type pill shape, we only cover 250 seconds, in order to provide a useful comparison due to its shorter oscillation period. The top row shows plots published by Männik *et al*. of the MinD maxima behavior and the bottom two rows show our simulations using the stochastic and deterministic models, respectively. We simulated approximations to the two shapes observed by Männik, which we call *shape A* and *shape B*. In addition, we studied two flattened stadium shapes which we call *stadium A* and *stadium B* corresponding in aspect ratio and thickness to the two experimental shapes. The spatial length scale of all the figures shown was identical. Each of the flattened cell shapes uses the same color scale for the number of proteins per unit area. Finally, we display the natural pill shape, which was also featured in Figs 2 and 3, with a different color scale to reflect the thicker cell containing more proteins per cross-sectional area.

As explained above, we focus on simulation of four illustrative flattened cell shapes: two shapes created to replicate those shapes observed experimentally by Männik *et al*.[13] (shape *A* and shape *B*), and for comparison two ‘stadium’ shapes that have the identical aspect ratio, thickness and volume (stadium *A* and stadium *B*), shown in Fig 4. All of the flattened cells have lost the rotational symmetry of the original pill shapes, but while shape *A* and shape *B* have only one mirror plane symmetry (that is normal to the flattened plane), stadium *A* and stadium *B* have three. This allows us to distinguish between the effect of flattening the cell and the cell shape’s irregularity and asymmetry.

From each of these flattened simulations, we have chosen a typical 350 second segment to compare with the published results of Männik *et al*.[13], exemplified in arrow plots in which the arrow heads show the location of sequential MinD maxima within the cell (in Fig 4). In addition to arrows between successive maxima in space and time, we plot as a colored background the density of MinD proteins averaged over the same time period. We note that we have manually verified that our (computer-generated) arrow plots also reflect a human interpretation of a movie of the same data. Finally, for comparison we present the same plot for a wild-type cell, with a time period of 250 seconds to account for its short period of oscillation.

In every case, including the wild-type pill shape, we see irregularity in the location of the maxima when using the stochastic model. The deterministic model shows uniformly bipolar oscillation. We conclude that this model is *not* chaotic (which would show irregularity in the deterministic computation), but rather that the irregularity results purely from stochastic processes. From these results and how they compare with experiment, we conclude that the deterministic model is inadequate to explain experimental observations of the locations of density maxima of the MinD protein. We note, however, that it is possible that there exists another deterministic model that does exhibit chaotic behavior.

The predictions of the stochastic model show some similarity to the experimental results in terms of irregularity of the locations of maxima, but the stochastic model displays significantly less variation in the direction of oscillations than experiment, suggesting inadequacies in the model. We also note that the stadium shapes in Fig 4 appear to have maxima location irregularity that is qualitatively similar to our irregular shapes: in both stochastic simulations the overall oscillation is largely bipolar, but with irregularity in the locations of the maxima that form at each end.

**Fig 5:**
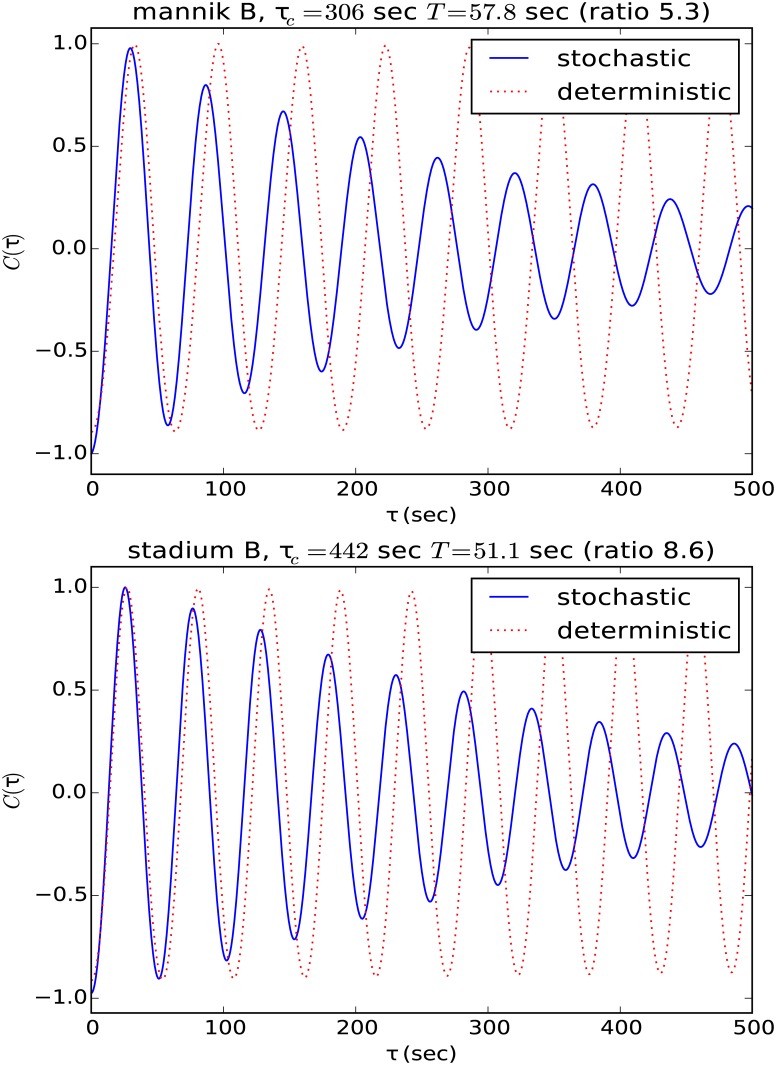
Temporal correlation function of the total MinD found in two opposite polar regions of the shape *B* (above) and the stadium *B* (below) cell shapes, shown against the correlation time. Data for both the the deterministic and stochastic models are shown, and the correlation functions are scaled to have the same initial value. For shape *B*, the stochastic model shows an oscillation period of 58 seconds and coherence time of 306 seconds, so that it takes roughly 5.3 periods for the behavior to decohere. For the stadium *B*, the same model shows an oscillation period of 51 seconds and coherence time of 442 seconds, so that it takes roughly 8.6 periods for the behavior to decohere.

However Fig 4 leaves unclear the importance of the flattening and accompanying lateral expansion of the cell versus cell shape irregularity and asymmetry in the temporal regularity of the oscillations. Although in the movies the stadiums appear to display, on average over long time scales, a somewhat more regular oscillation than appears in the irregular shapes *A* and *B*, the limited time range of the arrow plots (350 seconds) is inadequate to make this comparison. We therefore return to the correlation function between the number of proteins in a segment at the top of the cell and the number of proteins in a segment at the bottom of the cell. The “top” and “bottom” regions for computing the correlation time were established by dividing the long axis into thirds. This resulted in regions at the top and bottom ranging from 19% to 33% of the cell volume for shapes *A* and *B*, with the stadium shapes featuring 30% of the volume in the top and bottom regions. Fig 5 shows this plot for shape *B* and stadium *B*. Here we plot correlations of correlation times up to 500 seconds, a biologically relevant timescale. *E. coli* cell doubling times vary widely depending on environmental conditions, but will often be between 20 minutes to 100 minutes [37], while fluorescent microscopy experiments have shown that the FtsZ ring builds itself with a half life of roughly 30 seconds [38].

We see that both in the shape *B* and stadium *B*, the deterministic model is perfectly periodic. Both the deterministic and stochastic model correlations have the same number of proteins in their cells, and have been normalized with the same normalization factor, so that they can be directly compared against each other. The stochastic simulation of shape *B* has a coherence time of 5.3 periods, while the corresponding simulation of stadium *B* has a coherence time of 8.6 periods. The correlation function for stadium *A* is similar to that of stadium *B*, with similar coherence time (13 periods, not shown), while shape *A* also exhibits a similar coherence time (5.5 periods). This confirms that the stadium shapes do indeed oscillate somewhat more regularly than the irregular shapes, but both are comparable to the wild-type pill shape. From the stochastic correlation data, we conclude that irregularity of shape leads to a decrease in temporal regularity of the oscillatory behavior, although even the very distorted shape *B* exhibiting only a factor of two greater decoherence.

## Conclusion

We find qualitative agreement between our stochastic simulations and experiments showing disrupted bipolar MinD oscillation in asymmetric flattened *E. coli* cells [13]. As observed experimentally, our simulations predict that MinD maxima will form in a spatially irregular sequence of locations, which follow an overall bipolar trend. This result builds upon existing results showing that this model and its variants are effective in a variety of wild-type and mutated cell shapes [11, 12, 25], and reinforces that stochastic variations of Huang’s 2003 model [8, 11] has considerable predictive power beyond the standard wild-type cell.

In contrast to the stochastic model, the original deterministic version of the model [7] predicts behavior that contradicts experimental observations. Specifically, the deterministic model predicts robust and periodic bipolar oscillation in irregularly shaped cells. Thus we conclude that this method is inconsistent with experiment, and cannot be relied upon for predictions of the behavior of the MinD system. This is the most clear example of the inadequacy of the deterministic simulation method in reproducing experimental behavior of the MinD system to date. Until now the results of deterministic and stochastic simulation have largely coincided, with the stochastic method showing minor differences in behavior when compared to deterministic models [8, 11, 24, 25]. The regular oscillations in the deterministic case also allow us to conclude that Huang’s model is non-chaotic. Because of their similar construction, we anticipate that recent variants of the model [11, 20, 21] may also prove to be non-chaotic.

We find that flattened but regular and symmetric cells exhibit MinD oscillations that are qualitatively similar to the oscillations observed experimentally (and in our simulation) in irregular and asymmetric cells. This demonstrates that asymmetry is not required in order to induce spatially irregular MinD oscillations. In addition, the temporal regularity of oscillations is only moderately affected by irregularity in the shape of flattened cells. Thus we conclude that it may be the flattening of the cells, and the accompanying expansion in the transverse direction, that causes the disruption of regular bipolar oscillation which is observed by Männik *et al*.[13].

## Supporting Information

S1 Video Movie that shows the evolution of MinD density over a 250 second interval in a wild-type, pill-shaped cell. There is one frame for every half second of simulation time. Above are results from the stochastic model and below are results from the deterministic model. The density shown is integrated over the direction normal to the screen, so that the data is shown in molecules/*μ*m^2^.
(MP4)

S2 Video Movie that shows the evolution of MinD density over a 350 second interval in the shape A cell. There is one frame for every half second of simulation time. Above are results from the stochastic model and below are results from the deterministic model. The density shown is integrated over the direction normal to the screen, so that the data is shown in molecules/*μ*m^2^.
(MP4)

S3 Video Movie that shows the evolution of MinD density over a 350 second interval in the shape B cell. There is one frame for every half second of simulation time. Above are results from the stochastic model and below are results from the deterministic model. The density shown is integrated over the direction normal to the screen, so that the data is shown in molecules/*μ*m^2^.
(MP4)

S4 Video Movie that shows the evolution of MinD density over a 350 second interval in the stadium A cell. There is one frame for every half second of simulation time. Above are results from the stochastic model and below are results from the deterministic model. The density shown is integrated over the direction normal to the screen, so that the data is shown in molecules/*μ*m^2^.
(MP4)

S5 Video Movie that shows the evolution of MinD density over a 350 second interval in the stadium B cell. There is one frame for every half second of simulation time. Above are results from the stochastic model and below are results from the deterministic model. The density shown is integrated over the direction normal to the screen, so that the data is shown in molecules/*μ*m^2^.
(MP4)
